# How Should IgG Seroconversion to *Aspergillus fumigatus* be Interpreted in Children With Cystic Fibrosis

**DOI:** 10.1155/carj/9033903

**Published:** 2026-02-12

**Authors:** Hortense Petat, Damien Costa, Marc Lubrano, Laure Couderc, Christophe Marguet

**Affiliations:** ^1^ Department of Pediatrics, CHU Charles Nicolle, Univ Rouen Normandie, Dynamicure INSERM UMR 1311, Rouen, F-76000, France; ^2^ Laboratory of Parasitology-Mycology, EA7510 ESCAPE, CHU Charles Nicolle, Univ Rouen Normandie, Rouen, F-76000, France

**Keywords:** allergic bronchopulmonary aspergillosis, aspergillosis seroconversion, cystic fibrosis

## Abstract

**Background:**

Cystic fibrosis (CF) is one of the most common autosomal recessive diseases in the world population. Allergic bronchopulmonary aspergillosis (ABPA) is a severe complication of CF, the diagnosis of which is based on symptoms and blood IgE levels. However, many techniques of specific IgG levels’ measures are used, whose clinical significance is still unclear. We evaluated the clinical evolution of CF in children who presented a first *A. fumigatus* IgG seroconversion.

**Methods:**

Monocentric pediatric case‐control study led in Rouen, France. Every patient with a first *A. fumigatus* IgG seroconversion was paired with a seronegative patient. Clinical data, functional respiratory investigations, CT scan, and biologic data were collected a year before (*Y*_−1_), at the time of IgG seroconversion (*Y*_0_), and one year after (*Y*_+1_).

**Results:**

Thirty‐six cases were paired with 36 controls. Median age was 8. Forced expiratory volume in 1 s (FEV1) and forced vital capacity (FVC) were significantly lower at *Y*_+1_ (*p* = 0.025) and *Y*_0_ (*p* = 0.027), respectively, and more pulmonary exacerbations were observed in the case population (*p* = 0.047). Higher levels of specific IgE against *A. fumigatus* were observed at *Y*_−1_ (*p* = 0.001), *Y*_0_ (*p* = 0.014), and *Y*_+1_ (*p* = 0.04) in the case population. On the CT scan, bronchiectasis and pulmonary infiltrates were more frequent in the case population (*p* = 0.01 and *p* = 0.003, respectively).

**Conclusion:**

We found that the first *A. fumigatus* IgG seroconversion was associated with changes in clinical, respiratory functional, biologic, and radiologic parameters in CF pediatric population. *A. fumigatus* IgG seroconversion is an important step in the evolution of CF. A systematic search for seroconversion is therefore essential to assess the measures to be taken.

## 1. Introduction

Cystic fibrosis (CF) is one of the most common genetic disorders in the Caucasian population, with around 7740 patients actually registered in France. Allergic bronchopulmonary aspergillosis (ABPA) is defined by American and British diagnostic recommendations [1], and its prevalence can vary from study to study, ranging from 2.1% to 13.6% of the CF population worldwide [2, 3]. ABPA is most often diagnosed in CF patients over 6 years of age [4] and is associated with early colonization by *Pseudomonas aeruginosa* in sputum [5]. In patients with chronic *Pseudomonas aeruginosa* infection, the decline in respiratory function is greater if patients also have ABPA [5]. Thus, early detection is needed, and 5 stages are defined by Patterson [6] to guide therapeutic measures [1].

Diagnosis of ABPA still remains complicated [7]: diagnostic criteria associate clinical symptoms or/and radiologic abnormalities with biological markers. ISHAM‐ABPA working group recommended to diagnose ABPA in patients with predisposing conditions or compatible clinicoradiological presentation, with a mandatory demonstration of fungal sensitization and serum total IgE ≥ 500 IU·mL−1 and two of the following: fungal‐specific IgG, peripheral blood eosinophilia, or suggestive imaging [1]. However, these criteria are not very sensitive and specific [8]: in a systematic review published in 2023, the sensitivity for IgG diagnosing ABPA was found to be higher than IgE, while the specificity for IgE was higher [9]. Specific IgE, though interesting as a marker of ABPA [10] and recombinant IgE, could improve diagnosis sensitivity and specificity [11]. Af‐IgG4, thymus and activation‐regulated chemokine (TARC), and basophils stimulation tests have also been described as diagnostic markers [12, 13]. Although *Aspergillus fumigatus* IgG detection methods are quoted in the American CFF recommendations, their diagnostic value is often studied with the other ABPA markers. Lately, anti‐*Aspergillus* IgG presence has been considered as a diagnostic criterion of chronic bronchopulmonary aspergillosis [14]. Anti‐*Aspergillus* IgG appearance has rarely been associated with severe aspergillosis.

We propose here a retrospective study comparing both clinical and biological data of CF patients for whom a first *Aspergillus* seroconversion was noted. Clinical data, functional respiratory investigations, chest CT scans, and biologic data were collected one year before, at the time of IgG seroconversion, and one year after.

## 2. Materials and Methods

### 2.1. Patients

**Figure 1:**
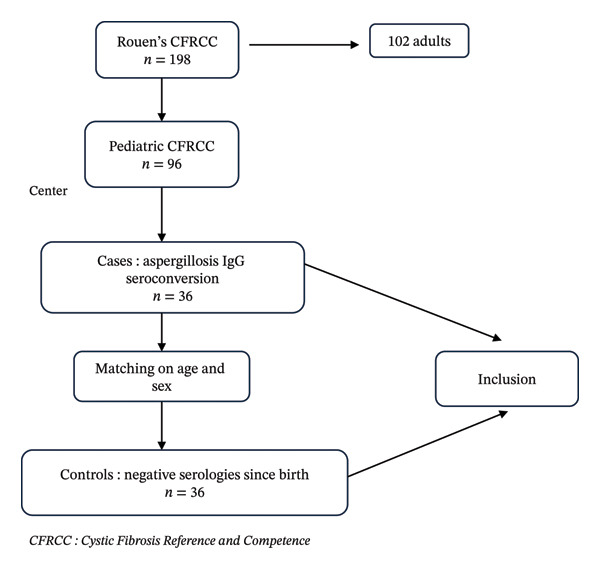
Flowchart of the population.

Inclusion criteria were as follows: patients < 18 years, followed in the pediatric department of Rouen Hospital and who benefited from systematic annual *A. fumigatus* IgG serology since CF diagnosis. Seroconversion was defined by a first positivity of serum anti‐*Aspergillus* IgG in patients who had never been negative. Detection of anti‐*Aspergillus* IgG was systematically evaluated using first Platelia *Aspergillus* IgG (Bio‐Rad) and next *Aspergillus* western blot IgG (LDBIO) kits according to manufacturer’s recommendations. Serologies were considered positive for anti‐*Aspergillus* IgG when both ELISA Platelia *Aspergillus* IgG and *Aspergillus* western blot IgG (simultaneous presence of at least 2 bands among P16, P18–20, P22, and P30) were positive. Each case was matched with a control (patient with CF with negative Af serologies) according to age and sex by a methodologist (Figure 1). No patient (case or control) met the diagnostic criteria for ABPA or *Aspergillus* infection.

### 2.2. Study Design

We conducted a monocentric, retrospective, pediatric, case‐control study, directed within the CF Reference and Competence Center (CFRCC) of Rouen, Normandy, France.

### 2.3. Study Endpoints

Demographic criteria, genetic criteria, and sweat test values were collected. Clinical status was determined a year before (*Y*_−1_), at the time of IgG seroconversion (*Y*_0_), and one year after (*Y*_+1_). CF patients were followed every 3 months at the hospital: a specialist medical consultation, sputum examination, and lung function tests (respiratory flow volumes and rates) if they were able to. The primary endpoint was the forced expiratory volume in 1 s (FEV1), in expected percentage of the age group, given by respiratory flow volumes and rates. Secondary endpoints were as follows: Body mass index (BMI), sweat test values at the diagnosis (mmol/L), forced vital capacity (FVC) (in expected percentage of the age group), total IgE levels (KUI/L), specific IgE levels (KUI/L), total IgG (g/L), serum eosinophils (cells/µL), *Pseudomonas aeruginosa* and *Staphylococcus aureus* quantitative colonization (CFU/mL), number of pulmonary exacerbations, antifungals prescription, and presence of bronchiectasis and pulmonary infiltrates on chest CT scans.

The number of pulmonary exacerbations was defined by the number of respiratory deteriorations which needed oral or intravenous antibiotics, leading or not leading to hospitalization. We also studied the frequency of antifungal administrations after positive serology and the duration of treatments. Bacteriological culture of sputum was systematically investigated in each annual evaluation. Chest CT scans were performed less frequently, so we collected the results of the CT scan performed closest to the time of seroconversion. They were analyzed by our pediatric radiologists, collecting the following criteria: bronchiectasis and pulmonary infiltrates.

### 2.4. Statistics

Statistics data were included in a Microsoft Excel table. Initial descriptive analysis was performed. Qualitative values were expressed in percentages or/and their effectiveness in the concerned population (%, n/N). Quantitative values were expressed with medians and interquartile ranges [1–3]. Data univariate analysis was made with statistics software (BIOSTATGV). Significance threshold was *p* ≤ 0.05. Mann–Whitney nonparametric tests were used for quantitative comparisons. Chi^2^ and Student’s tests were used for qualitative and quantitative comparisons, respectively. Data bivariate analysis was made with statistics software (BIOSTATGV). Significance threshold was *p* ≤ 0.05.

### 2.5. Ethics Approval and Consent to Participate

Parents of patients were informed and consented to the publication of the cases. For the ethics committee, ethical approval was granted by the local committee for ethics in health research at the University Hospital of Rouen.

## 3. Results

We included 36 patients in each group, with a median age of 8 years [IQ_25–75_: 5–11]. None of the patients met the diagnostic criteria for ABPA. BMI, sweat test values, and F508del homozygote genotypes were not significantly different in each group (Table 1). We analyzed lung function of 25 children paired, because the other were too young. No significant difference was observed in FEV1 at *Y*_−1_ and *Y*_0_ (Table 2). FEV1 was significantly lower at *Y*_+1_ in the cases compared to the controls (*p* = 0.025). FVC was significantly lower at *Y*_0_ in the cases compared to controls (*p* = 0.027). Pulmonary exacerbations between *Y*_−1_ and *Y*_+1_ were significantly more frequent in the cases (*p* = 0.047) with more than 3 exacerbations during the period, versus 1.5 in the control group. The number of patients treated with antifungals and the duration of treatment are noted in Table 3. Among treatments, itraconazole was prescribed in 71% of the cases and voriconazole in 27% of the cases.

**Table 1:** Characteristics of populations.

	**Cases *n *= 36**	**Controls *n *= 36**	**p**

Sweat test value (mmol/L)	97 (79; 111)	92 (69; 105)	0.25
F508del homozygote genotype *n* (%)	16 (44)	13 (36)	0.63
BMI (percentile of age)	45 (24; 67)	40 (18; 51)	0.40

*Note:* Results are expressed with median, [25%–75%] interquartile.

**Table Table​ 2:** Evolution between Y−1 and Y+1.

	**Cases**			**Controls**		
	**Y**_−1_	**Y**_0_	**Y**_+1_	**Y**_−1_	**Y**_0_	**Y**_+1_
Exacerbations (n)	3 (2–4.5)^1^			1.5 (0.75–3)^1^		
FEV1 (% expected value) *n *= 50	103	93	921	94	93	101^1^
	(89.5–106.5)	(78–105)	(77–104)	(82.5–112)	(89–107)	(96–121)

FVC (% expected value) *n *= 50	93	90^1^	94^1^	97	101^2^	100^1^
	(85–102)	(78–105)	(82–106)	(85–99)	(89–107)	(89–113)

*Note:* Results are expressed with median, [25%–75%] interquartile. All values are results of the cases compared with witnesses.

^1^ *p* < 0.05.

^2^ *p* < 0.001.

**Table Table​ 3:** Characteristics at Y0.

	**Cases**	**Controls**

Blood eosinophils (cells/µL)	200 (120; 850)	240 (90; 420)
PA colonization, *n* (%)	5 (13.8)	3 (8.9)
SA colonization, *n* (%)	26 (72.3)	26 (72.3)
CT scan	*n *= 32	*n *= 28
Presence of infiltrates *n* (%)	12 (37.5)^2^	1 (4)^2^
Presence of bronchiectasis *n* (%)	23 (72)^1^	11 (36)^1^
Antifungals (n)	24	3
Duration (months)	13 (8–15)	10 (16–24)

*Note:* Results are expressed with median, [25%–75%] interquartile. All values are results of the cases compared with witnesses.

Abbreviations: PA, *Pseudomonas aeruginosa*; SA, *Staphylococcus aureus*.

^1^ *p* < 0.05.

^2^ *p* < 0.001.

With regard to total IgE (Figure 2), no significant differences between cases and controls were observed over time. Significant increases in specific IgE at *Y*_−1_ (*p* = 0.001), *Y*_0_ (*p* = 0.014), and *Y*_+1_ (*p* = 0.029) were observed in the cases compared to the controls. Total IgG was significantly higher at *Y*_0_ (*p* = 0.04) in the cases compared to the controls, and not at *Y*_−1_ and *Y*_+1_. No significant difference was shown in serum eosinophils (*p* = 0.87) and chronic colonizations by *Pseudomonas aeruginosa* (*p* = 0.72) and *Staphylococcus aureus* (*p* = 1) at Y0 in secretions between cases and controls. Every chest CT scan was performed in the systematic follow‐up of patients, never in an emergency situation, in 32 cases and 28 controls. More bronchiectasis (*p* = 0.014) and more infiltrates (*p* < 0.001) were significantly described by the radiologists in the case group compared to controls (Table 3).

Figure 2Evolution of total IgE (a), specific IgE (b), and total IgG (c) the year before (*Y*_−1_), at the moment (*Y*_0_), and the year after (*Y*_1_) IgG seroconversion.

**Figure:**
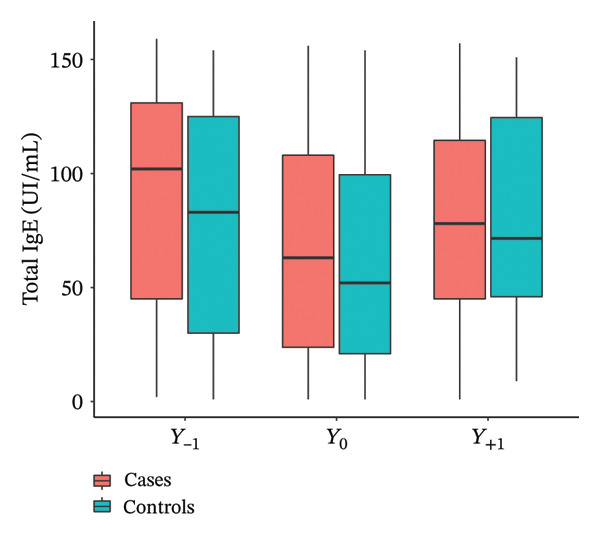
(a)

**Figure:**
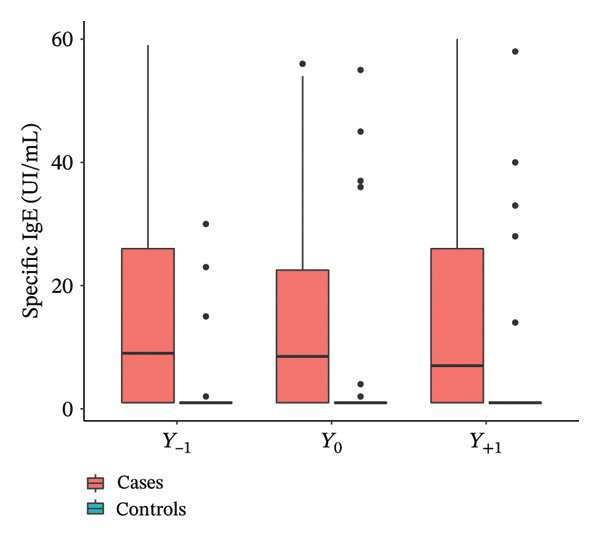
(b)

**Figure:**
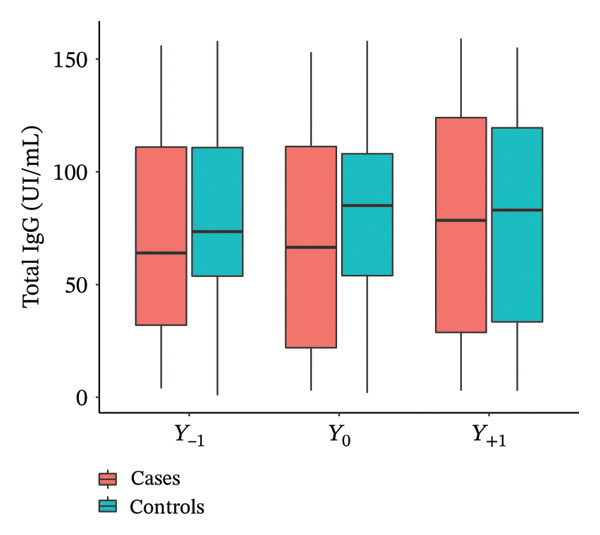
(c)

## 4. Discussion

ABPA is the expression of aspergillosis in CF with various clinical presentations [15] associated with variable markers and biological thresholds according to the different recommendations. Diagnosis of ABPA is still a challenge for the clinicians. We report here a retrospective case‐control study reporting *A. fumigatus* IgG seroconversion forms in pediatric patients with CF. The main results showed greater impairment of FEV1, more exacerbations, and higher levels of specific IgE in the case population.

FEV1, as the primary endpoint, was analyzed in patients paired on age and sex (22 patients were too young to have FEV1 data). FEV1 and FVC were significantly decreased a year after *A. fumigatus* IgG seroconversion in the cases compared to controls. In the European study [2], no relation between FEV1 decrease and ABPA was observed, highlighting heterogeneous evolution within a big cohort.

Interestingly, patients with seroconversion have significantly more pulmonary exacerbations. We only noted exacerbations requiring antibiotics; this cannot be explained by more frequent infections by *Pseudomonas aeruginosa* or *Staphylococcus aureus*, because colonization and chronic infections were similar in the two groups. A higher frequency of chronic infections by *Pseudomonas aeruginosa* could be expected [4]. However, this result goes in the same direction as the FEV1 decrease observed in the seroconversion group: Exacerbation frequency is associated with a higher FEV1 decrease. Concerning the possible role of *Aspergillus fumigatus* sensitization, in previous studies, a FEV1 decrease was noted in patients sensitized at *Aspergillus fumigatus* in CF [11] and also asthma [12].

The third point of our study is the observation of the most important bronchial disease, with bronchiectasis, which is twice as important in the cases as in the controls. We noted a parenchymal disease in 1 child of the control and in 33% cases. Bronchiectasis is one of lesional damages caused by CF, and infiltrates belong to ABPA criteria. Our results, therefore, show that children with seroconversion appear to progress to more severe disease.

Baxter proposed an immunologic classification of ABPA [16], with total and specific IgG and IgE, galactomannan dosage, and *Aspergillus fumigatus* detection performed by RT‐PCR. Four groups were defined as follows: the first without aspergillosis but which can be a healthy carrier; the second, positive for the whole markers corresponding to real ABPA; the third, which is a group of patients sensibilized with low IgG rate; and the fourth corresponding to patients with high IgG levels and no IgE and a high galactomannan level, defined as *Aspergillus* bronchitis. Patients with ABPA and sensitized were more allergic. So, analysis of our patients with seroconversion showed that 25 patients (70%) were sensitized with specific IgE > 0,1 UI/L, 9 with total IgE > 100 KUI/L, and 1 with ABPA. However, among 11 with seroconversion, only 1 became sensitized a year after, showing a possible dissociation between IgG and IgE productions, compatible for an *Aspergillus* bronchitis. Furthermore, our cases showed more bronchial involvement, suggesting a potential onset of *Aspergillus* bronchitis. In ABPA associated with CF, elevated *Aspergillus*‐specific IgG reflects chronic exposure and immune response to the fungus, supporting the diagnosis but not replacing IgE as the main marker of allergic sensitization. High IgG levels help distinguish ABPA from other *Aspergillus-*related lung diseases and indicate ongoing or past fungal colonization. However, IgG is less useful for monitoring acute disease activity compared to IgE.

All these points have therapeutic implications. Two major treatments are routinely used in ABPA cases: oral corticosteroids and antifungals. Sixty‐seven percent of cases have been treated with antifungal agents for a median period of 11.4 months. Itraconazole was the first prescribed. Voriconazole was prescribed more rarely if the serology stayed positive with itraconazole. No patient received corticosteroids. These first positive serologies were not considered as real ABPA, but as a potential ABPA beginning. By showing a more marked clinical course in cases, we can suggest prescribing earlier treatment of *A. fumigatus* IgG seroconversions.

## 5. Conclusion

The results of our case‐control study show clinical, functional, and injury modifications by patients presenting with a first IgG‐*Aspergillus* seroconversion. These are in favor of the early effect of aspergillosis colonization and of the interest of following children regularly. The discussion of early treatment of these forms seems to be a major issue, the aim being to limit negative clinical evolution as much as possible.

**Nomenclature**
ABPA: Allergic bronchopulmonary aspergillosis
Af: *Aspergillus fumigatus*
BMI: Body mass index
CF: Cystic fibrosis
CFF: Cystic Fibrosis Foundation
FEV1: Forced expiratory volume in 1 s
FVC: Forced vital capacity

## Author Contributions

Hortense Petat: literature search, data collection, study design, analysis of data, and manuscript preparation.

Damien Costa: study design and analysis of data.

Laure Couderc and Marc Lubrano: data collection and review of the manuscript.

Christophe Marguet: study design and review of the manuscript.

## Funding

This research did not receive any specific grant from funding agencies in the public, commercial, or not‐for‐profit sectors.

## Disclosure

This study was published as a preprint (https://www.authorea.com/users/656963/articles/662005-first-aspergillus-fumigatus-igg-seroconversion-is-associated-with-more-severe-disease-in-cf-children?commit=8062e28abd95fb15cd25e6ad1f6bdaf07b95e94f).

## Conflicts of Interest

The authors declare no conflicts of interest.

## Data Availability

The data that support the findings of this study are available on request from the corresponding author. The data are not publicly available due to privacy or ethical restrictions.
